# Acoustic-transfection for genomic manipulation of single-cells using high frequency ultrasound

**DOI:** 10.1038/s41598-017-05722-1

**Published:** 2017-07-13

**Authors:** Sangpil Yoon, Pengzhi Wang, Qin Peng, Yingxiao Wang, K. Kirk Shung

**Affiliations:** 1 Department of Biomedical Engineering, University of Southern California, 1042 Downey Way, Los Angeles, California, 90089 USA; 2 Department of Bioengineering & Institute of Engineering in Medicine, University of California, San Diego, 9500 Gilman Drive, La Jolla, California, 92093 USA

## Abstract

Efficient intracellular delivery of biologically active macromolecules has been a challenging but important process for manipulating live cells for research and therapeutic purposes. There have been limited transfection techniques that can deliver multiple types of active molecules simultaneously into single-cells as well as different types of molecules into physically connected individual neighboring cells separately with high precision and low cytotoxicity. Here, a high frequency ultrasound-based remote intracellular delivery technique capable of delivery of multiple DNA plasmids, messenger RNAs, and recombinant proteins is developed to allow high spatiotemporal visualization and analysis of gene and protein expressions as well as single-cell gene editing using clustered regularly interspaced short palindromic repeats (CRISPR)-associated protein-9 nuclease (Cas9), a method called acoustic-transfection. Acoustic-transfection has advantages over typical sonoporation because acoustic-transfection utilizing ultra-high frequency ultrasound over 150 MHz can directly deliver gene and proteins into cytoplasm without microbubbles, which enables controlled and local intracellular delivery to acoustic-transfection technique. Acoustic-transfection was further demonstrated to deliver CRISPR-Cas9 systems to successfully modify and reprogram the genome of single live cells, providing the evidence of the acoustic-transfection technique for precise genome editing using CRISPR-Cas9.

## Introduction

Intracellular delivery of macromolecules into target cells is an essential and fundamental procedure to modulate cell functions for research and clinical applications. Intracellular delivery of a wide variety of macromolecules such as DNA plasmids and messenger RNA (mRNA), as well as irregular materials such as proteins, increases the selection options among various types of materials when controlling the activity of cells for diagnosis and therapeutic purposes^1, 2^. Simultaneous intracellular delivery of multiple macromolecules further provides a new opportunity for precise gene editing using CRISPR-Cas9^3–5^ and the generation of induced pluripotent stem cells (iPSCs) by using multiple reprogramming factors^6^. A single-cell level intracellular delivery provides a selective and targeted introduction of different fluorescence resonance energy transfer (FRET)-based biosensors into physically connected neighboring cells to reveal unique cell-to-cell interactions by live cell imaging with high spatiotemporal resolutions^7^. Here we describe a versatile intracellular delivery technique, a method called acoustic-transfection, which can noninvasively and remotely deliver diverse macromolecules simultaneously or sequentially without microbubbles. Ultrasound allows for manipulation at the single-cell (micrometer) level and deep tissue or organ (millimeter) level depending on center frequency^8, 9^. High frequency ultrasound, with a center frequency of over 150 MHz, focuses acoustic energy into a diameter of 10 μm or less. High frequency ultrasound is also unlikely to induce cavitation, which is the main mechanism of low frequency ultrasound and contrast agents (microbubble) based sonoporation^10, 11^.

Among the currently available delivery methods, viral-vectors, nanoparticle- and lipid-based delivery techniques rely on vesicles that carry macromolecules, which intrinsically lack specificity in spatial targeting^12, 13^. Viral-vectors are highly efficient but they can integrate into the host genome, thereby increasing the possibility of tumorigenesis^12^. Moreover, it is difficult for viral-vectors to deliver non-genetic molecules and simultaneous delivery of different species of molecules using viral-vectors is challenging. After endocytosis of nanoparticles and liposomes, endosomal escape remains a question, which limits the efficiency of nanoparticle- and lipid-based delivery techniques^14^. Generating pores on cell membranes through physical deformation is another way to deliver desired macromolecules. Microinjection, electroporation, microfluidics with constriction, optoporation^15^ and sonoporation fall into this category^16–19^. Microinjection may be used for a very efficient way of delivering macromolecules into cells; however, equipment for microinjection is usually too expensive and requires highly skilled personnel^16^. Electroporation has a high cytotoxicity and the associated electrical fields may affect both the cells and the macromolecules. Microfluidics with constriction lacks the capability of single-cell level targeting to specifically control cell-pairs for cell-to-cell interactions^18^. Optoporation may induce cell death by exposing excessive laser energy to target cells. Low frequency ultrasound-based sonoporation is more suitable for *in vivo* applications due to its deeper penetration depth. However, sonoporation depends significantly on the cavitation of microbubbles to increase the permeability across the cell lipid bilayer^11^. The concern is that cavitation may result in damage and non-specific alterations to targeted cells^10^.

**Figure 1:**
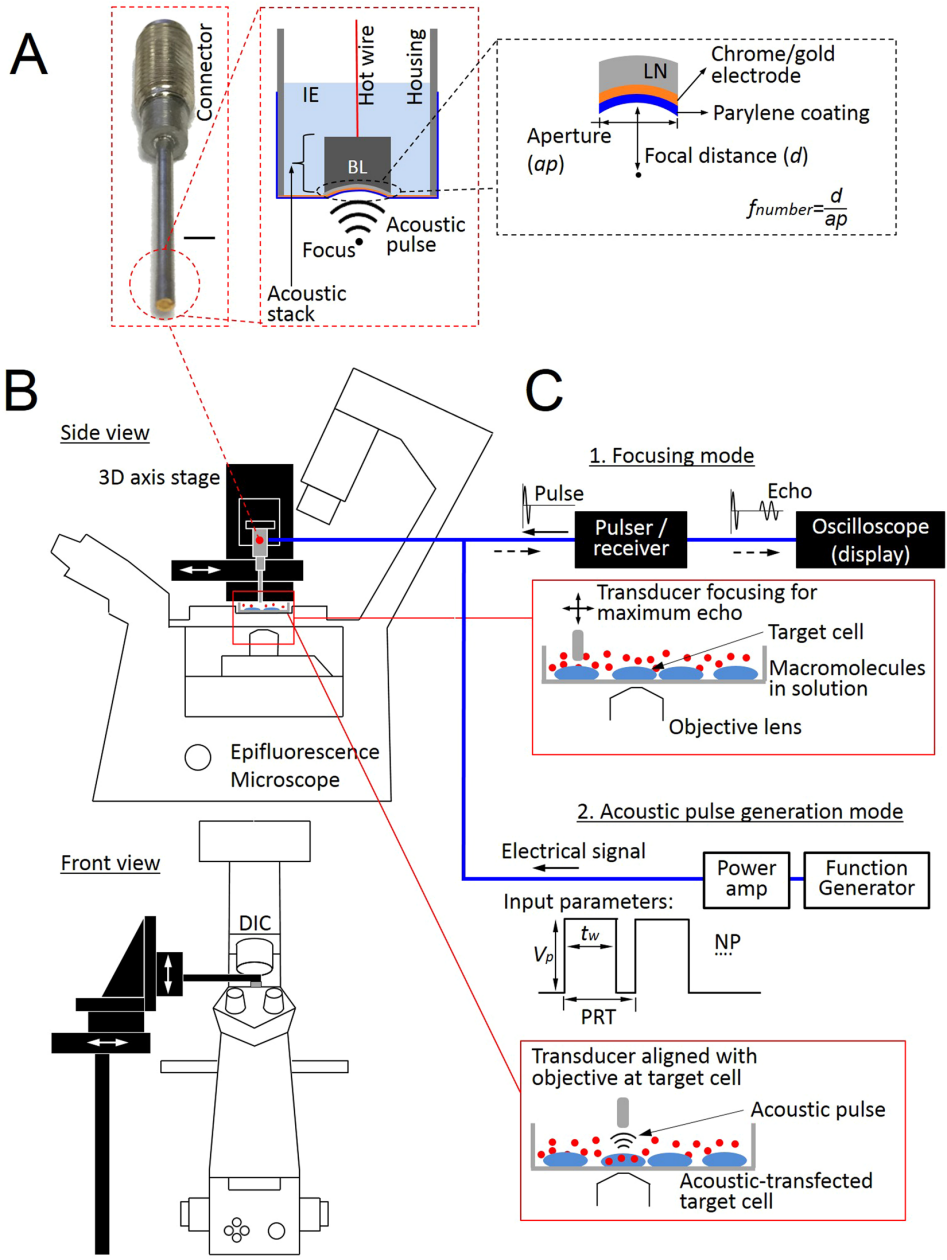
Acoustic-transfection system. (**A**) Schematic diagram of an acoustic-transfection system and a high frequency ultrasonic transducer. (**B**) A 3D axis stage was integrated with epifluorescence microscope to precisely control the location of a high frequency ultrasonic transducer (grey color). The high frequency ultrasonic transducer was mounted on a 3D axis stage. The 3D axis moves in the x, y, and z directions in 0.3 μm increments. A petridish that contained cells and solution with macromolecules were placed on the epifluorescence microscope stage for acoustic-transfection. (**A**) A photograph of a high frequency ultrasonic transducer, used in this study, is shown in the first dashed rectangle (Scale bar, 2 mm). An acoustic stack consisting of a conductive backing layer, BL, and lithium niobate, LN, generated an acoustic pulse to treat cells by the excitation of an electrical signal, transmitted through hot wire. The aperture, *ap*, is the diameter of the LN. The acoustic stack was placed at the tip of the housing and fixed with insulating epoxy, IE, and spherically focused to a focal distance, *d*. The chrome/gold layer in front of the LN is for the ground connection. A parylene coating is used for protection from corrosion. (**C**) A target single-cell for acoustic-transfection is selected by the epifluorescence microscope using an appropriate objective lens. The ultrasonic transducer was connected to a pulser/receiver and an oscilloscope (display) to place the focus of the ultrasonic transducer at the target single-cell. A pulser/receiver excited the ultrasonic transducer by transmitting a pulse. The reflected echo signal from the bottom of the petridish was detected by the same transducer and amplified by the same pulser/receiver. The maximum echo signal on the oscilloscope (display) indicated that the focus of the ultrasonic transducer was located properly for acoustic-transfection. By moving the ultrasonic transducer horizontally, the ultrasonic transducer was aligned with the objective lens for acoustic-transfection and monitoring of the target single-cell. When in the acoustic pulse generation mode, the ultrasonic transducer was connected to a function generator and a power amplifier. An electrical signal was generated by a function generator and amplified by a power amplifier to excite the transducer to generate the acoustic pulses for acoustic-transfection. Acoustic pulses can be precisely controlled using a function generator to maintain input parameters including *Vp*, *tw*, PRT, and NP.

In our previous work, we optimized the input parameters such as peak-to-peak voltage (*Vp*), pulse width (*tw*), pulse repetition time (PRT), and the number of pulses (NP) (Fig. 1C2) using FRET-based Ca^2+^ biosensors for safe and efficient intracellular delivery conditions^20^. We also demonstrated a potential for intracellular delivery of small exogenous molecules such as propidium iodide (MW: 0.7 kDa) and 3 kDa dextran using acoustic-transfection^20^. However, these delivered compounds are relatively inert and do not allow the manipulation of target cells. In the current paper, we demonstrate the intracellular delivery of DNA plasmids, mRNAs, recombinant proteins, and CRISPR-Cas9 systems using acoustic-transfection to visualize and genetically edit cells by homologous-directed repair (HDR). This demonstration proves that acoustic-transfection can deliver a wide range of biologically active macromolecules noninvasively and remotely by utilizing high frequency ultrasound. Acoustic-transfection can be directly and easily controlled using electrical signals as the input parameters since the use of microbubbles is not required. In addition, acoustic-transfection directly applies acoustic pulses to deliver active macromolecules (Fig. 1C2). The results show potential advantages over other transfection techniques when studying cell-to-cell interaction by combining acoustic-transfection with FRET-based biosensors (BS) and live cell imaging techniques to visualize important molecular events and cell reprogramming by repeatedly delivering multiple recombinant proteins or CRISPR-Cas9^5, 6^.

## Results

### Delivery efficiency and gene expression depending on key factors

**Figure 2:**
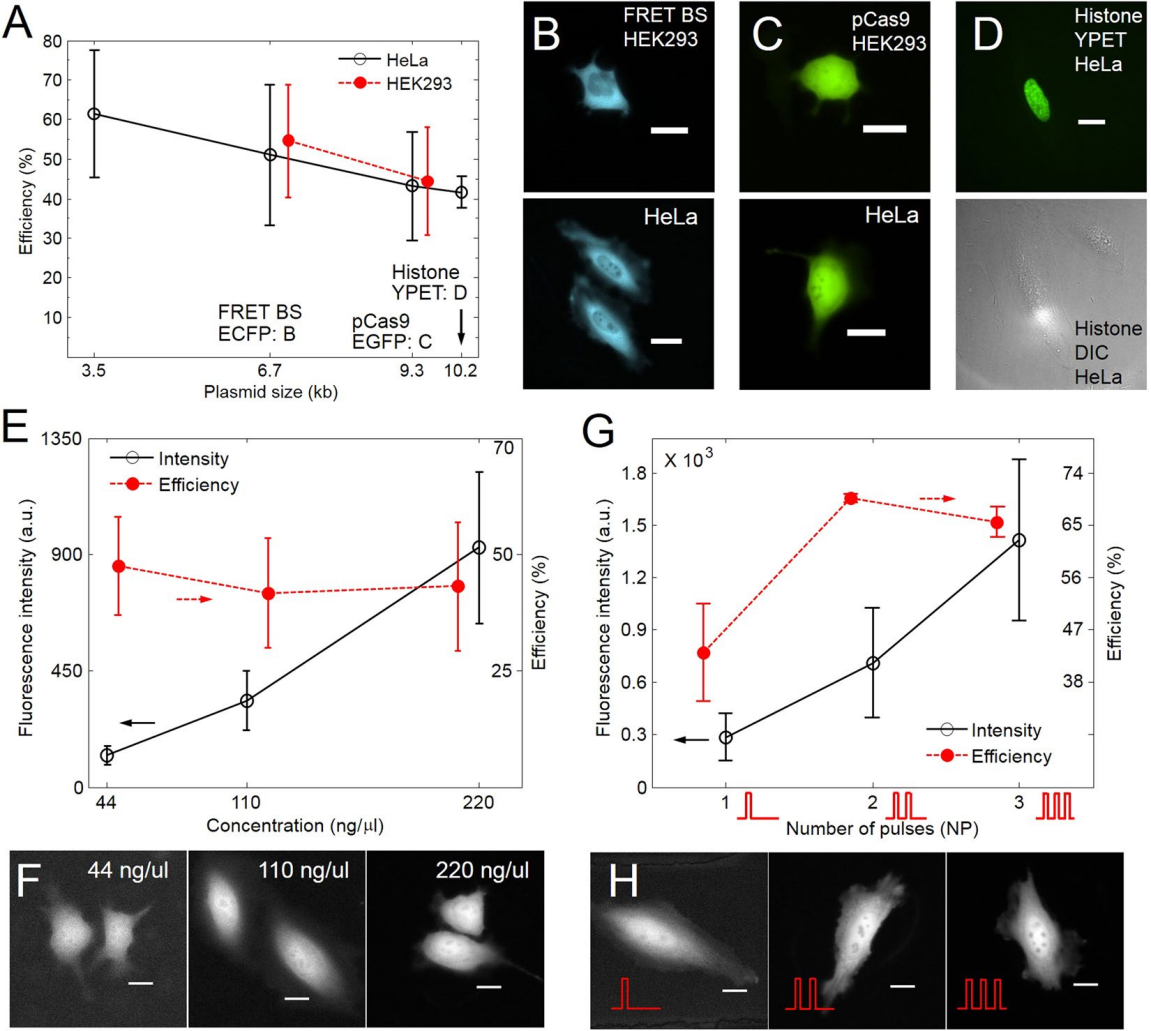
Gene expression level (fluorescence intensity) and delivery efficiency of acoustic-transfection depending on plasmid size and concentration as well as the NP. The *Vp* and *tw* of each electrical pulse was 22 V and 18 μs, respectively and the NP was 1 unless otherwise indicated. For the NP = 2 and NP = 3 cases, the PRT was 0.5 s. (**A**) pMax-EGFP (3.5 kb), FRET-Ca^2+^-biosensor (BS) with ECFP (6.7 kb), pCas9-EGFP (9.3 kb), and Histone-H3-targeted-YPET (10.2 kb) plasmids were used. The concentration of plasmid was 110 ng/μl. For the HeLa cells, each data point was run two times and each run was performed on 6 to 21 cells. For HEK293 cells, each data point was run three times and each run was performed on 10 to 38 cells. Representative fluorescence images of the acoustic-transfected HEK293 (upper row) and HeLa (lower row) cells with (**B**) FRET-Ca^2+^-BS-ECFP and (**C**) pCas9-EGFP are shown. (**D**) Fluorescence (upper low) and DIC (lower row) images of the HeLa cells, acoustic-transfected with Histone-H3-targeted-YPET plasmids, is presented. The pCas9-EGFP and HeLa cells were used for (**E**–**H**). (**E**) For efficiency calculation, two independent experiments were performed at each data point and 17 to 21 cells were used for each experiment. Eight cells were randomly chosen and the fluorescence intensity was estimated. (**F**) Representative fluorescence images show the pCas9-EGFP expression after 24 hours under the concentrations of 44 ng/μl, 110 ng/μl, and 220 ng/μl. (**G**) Two independent experiments were run for the NP = 1, 2, and 3 cases with a concentration of 110 ng/μl. Each experiment used 16 to 21 cells. Thirteen cells were randomly chosen for fluorescence intensity calculation. (**H**) Representative fluorescence images of the pCas9-EGFP after 24 hours for the cases of NP = 1, 2, and 3 are shown. Error bars represents +/− one standard deviation (SD). Scale bars indicate 20 μm.

Because DNA plasmids are negatively charged, relatively large, and stable, we chose DNA plasmids for our studies regarding delivery efficiency and gene expression. We hypothesized that the size and concentration of DNA plasmids as well as the number of pulses (NP, Fig. 1C2) should affect delivery efficiency and gene expression after acoustic-transfection. Key factors were defined as the size and concentration of the DNA plasmids as well as the NP. The dependency of the delivery efficiency on DNA size was investigated using HeLa cells, pMax-EGFP (3.5 kb), FRET-BS-CyanYPET (6.7 kb), pCas9-EGFP (9.3 kb), and Histone-H3-targeted-YPET (10.2 kb). To confirm delivery efficiency of a different cell line, we further performed acoustic-transfection of FRET-BS-CyanYPET and pCas9-EGFP vectors using HEK293 cells. As we expected, the delivery efficiency is robust among the different DNA plasmids under the same acoustic-transfection conditions (Fig. 2A). Representative fluorescence images of gene expression of the ECFP by FRET-BS-CyanYPET, EGFP by pCas9, and YPET by Histone-H3-targeted plasmids are shown in Fig. 2B–D, respectively. HEK293 cells exhibit slightly higher delivery efficiency, but the difference is not statistically significant.

The pCas9-EGFP plasmid was chosen for subsequent experiments because it is one of the components of the CRISPR-Cas9 and has a relatively large size. Transient gene expression after transcription and translation depends on the initial intracellular delivery dosage of DNA plasmids. Gene expression level (fluorescence intensity) of the delivered pCas9-EGFP increases due to an increased copy number of delivered DNA plasmids (Fig. 2E). A stronger gradient of DNA plasmid concentration across the cell membrane drives stronger intracellular transport of pCas9-EGFP plasmids. Figure 2F shows the representative EGFP expression depending on the concentrations of pCas9 plasmid. Unless otherwise noted, we used 110 ng/μl for all DNA plasmid acoustic-transfection experiments afterwards in the manuscript. Gene expression of pCas9-EGFP in HeLa cells was monitored up to 21 hours under an epifluorescence microscope. The cells functioned normally and daughter cells had the same gene expression (Supplementary Video 1).

The NP directly affects both the delivery efficiency and gene expression level (fluorescence intensity) as shown in Fig. 2G. Consecutive pulses were applied every 0.5 seconds for an NP = 2 and NP = 3. Gene expression (fluorescence intensity) increased in proportion to the NP, which was expected because the NP was directly related to the delivered copy number of pCas9-EGFP and its efficiency. Efficiency of delivery increased approximately 50% when the NP changed from 1 to 2 but did not change further significantly after one more acoustic pulse (e.g., NP = 3) was applied. EGFP fluorescence images in Fig. 2H represent different levels of gene expressions for NP = 1, 2 and 3. The results indicate that acoustic-transfection efficiency and gene expression (fluorescence intensity) clearly depend on key factors of acoustic-transfection and the relationship is predictable.

### Sequential and simultaneous single-cell level delivery of nucleic acids

**Figure 3:**
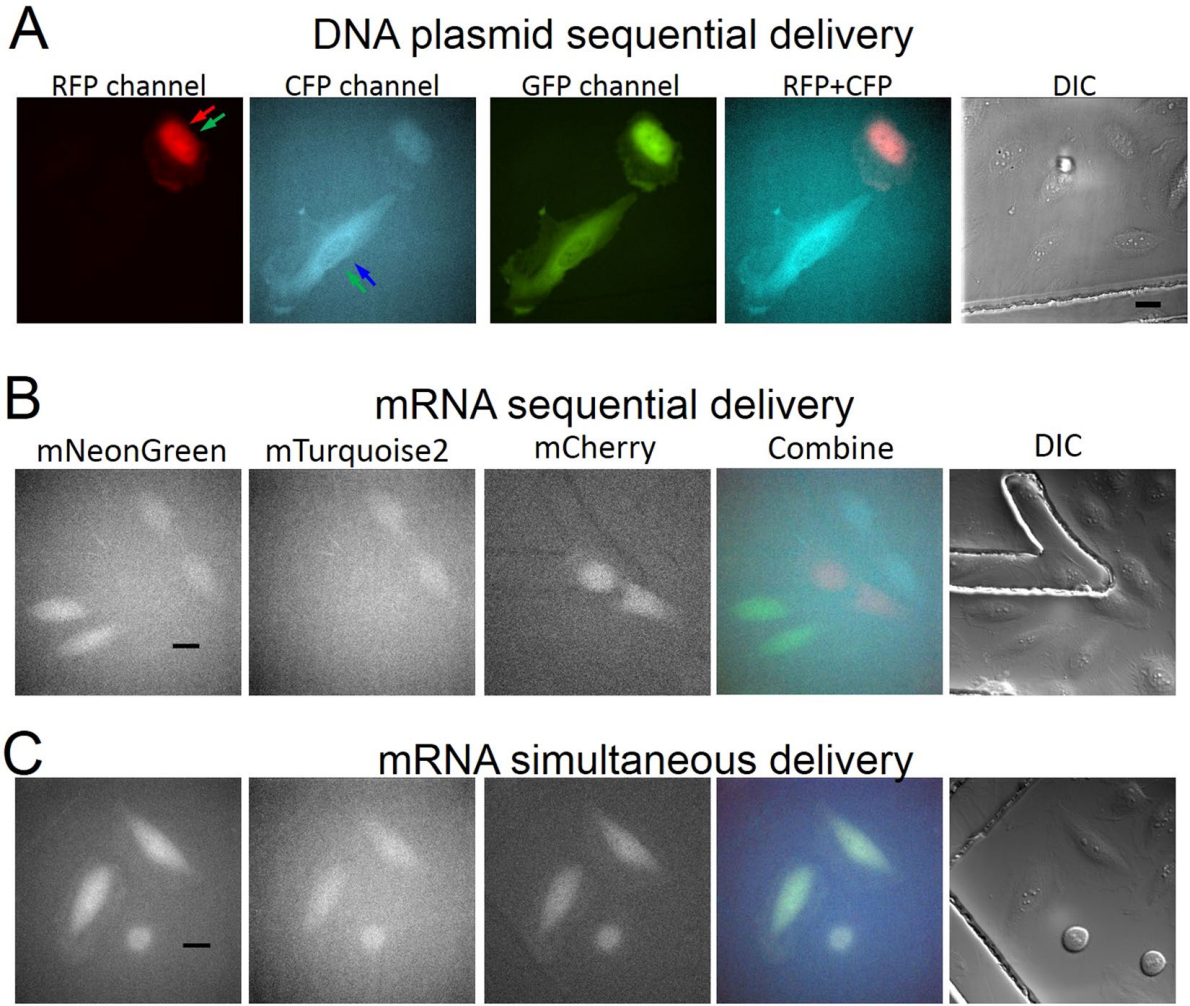
Sequential and simultaneous single-cell intracellular delivery of DNA plasmids and mRNA into adjacent cells. The *Vp* and *tw* of each electrical pulse was 22 V and 18 μs, respectively, and NP = 1 unless otherwise indicated. (**A**) Green and red arrows in the first panel and blue and green arrows in the second panel indicate that the cells were acoustic-transfected with FRET-BS-GreenRed and FRET-BS-CyanYPET, respectively. Images obtained by RFP (first panel) and CFP (second panel) channels show two adjacent cells with RFP and ECFP fluorescence by FRET-BS-GreenRed and FRET-BS-CyanYPET plasmids. (**B**) The mRNA strands expressing mNeonGreen (mNG), mTurquoise2 (mTQ2), and mCherry (mCR) were sequentially transfected into individual neighboring cells. Images from left to right were obtained by GFP, CFP, and RFP channels after 20 hours of acoustic-transfection of three types of mRNA. Fluorescence images of the same cells after 40 hours are also available, as shown in Supplementary Fig. 6A. (**C**) Simultaneous delivery of the mNG, mTQ2, and mCR mRNAs was also successfully demonstrated. Three adjacent cells were targeted and three cells functioned normally, thereby indicating the low cytotoxicity of acoustic-transfection. Scale bars indicate 20 μm.

An ECFP and YPET expressing FRET- BS-CyanYPET^7^ and EGFP and RFP expressing FRET-BS-GreenRed pair were separately delivered into adjacent individual cells to demonstrate the sequential single-cell intracellular delivery via acoustic-transfection. One cell was initially acoustic-transfected with FRET-BS-GreenRed. After washing with Hank’s balanced salt solution (HBSS) medium, a cell next to the previously transfected cell was acoustic-transfected with FRET-BS-CyanYPET. Targeted cells were revisited for imaging after 15 hours where they expressed GFP/RFP and CFP/YPET fluorescence as designed and shown in Fig. 3A. Red channel image clearly indicates that acoustic-transfection specifically targeted a single cell and transfected FRET-BS-GreenRed in Fig. 3A. There is no red fluorescence in adjacent cells. Both cells have GFP fluorescence under GFP channel image because of YPET and GFP fluorophores. Weaker CFP fluorescence of a FRET-BS-GreenRed transfected cell in CFP channel image is observed due to bleed-through between CFP and GFP channels. Intracellular calcium cell signaling among adjacent cells, acoustic-transfected with different FRET biosensors, can be visualized using sequential delivery.

As part of the experimental process, mRNA strands encoding mNeonGreen, mTurquoise2, and mCherry were generated and sequentially delivered into different cells (details in Materials and Methods). Acoustic-transfected cells were imaged using GFP, CFP, and RFP channels of an epifluorescence microscope 20 hours after transfection as shown in Fig. 3B. After 40 hours of acoustic-transfection, these same cells were examined again, displaying the same fluorescence (Supplementary Fig. 6A). Simultaneous intracellular delivery of three types of mRNAs into three adjacent cells was performed. The acoustic-transfected cells showed GFP, CFP, and RFP fluorescent signals in the same cells 20 hours and 40 hours after transfection (Fig. 3C and Supplementary Fig. 6B). Due to filter settings in microscope described in Materials and Methods, EGFP signal in the second panel of Fig. 3A and mTurquoise2 signal in the first panel of Fig. 3B are observed.

### Recombinant protein delivery

**Figure 4:**
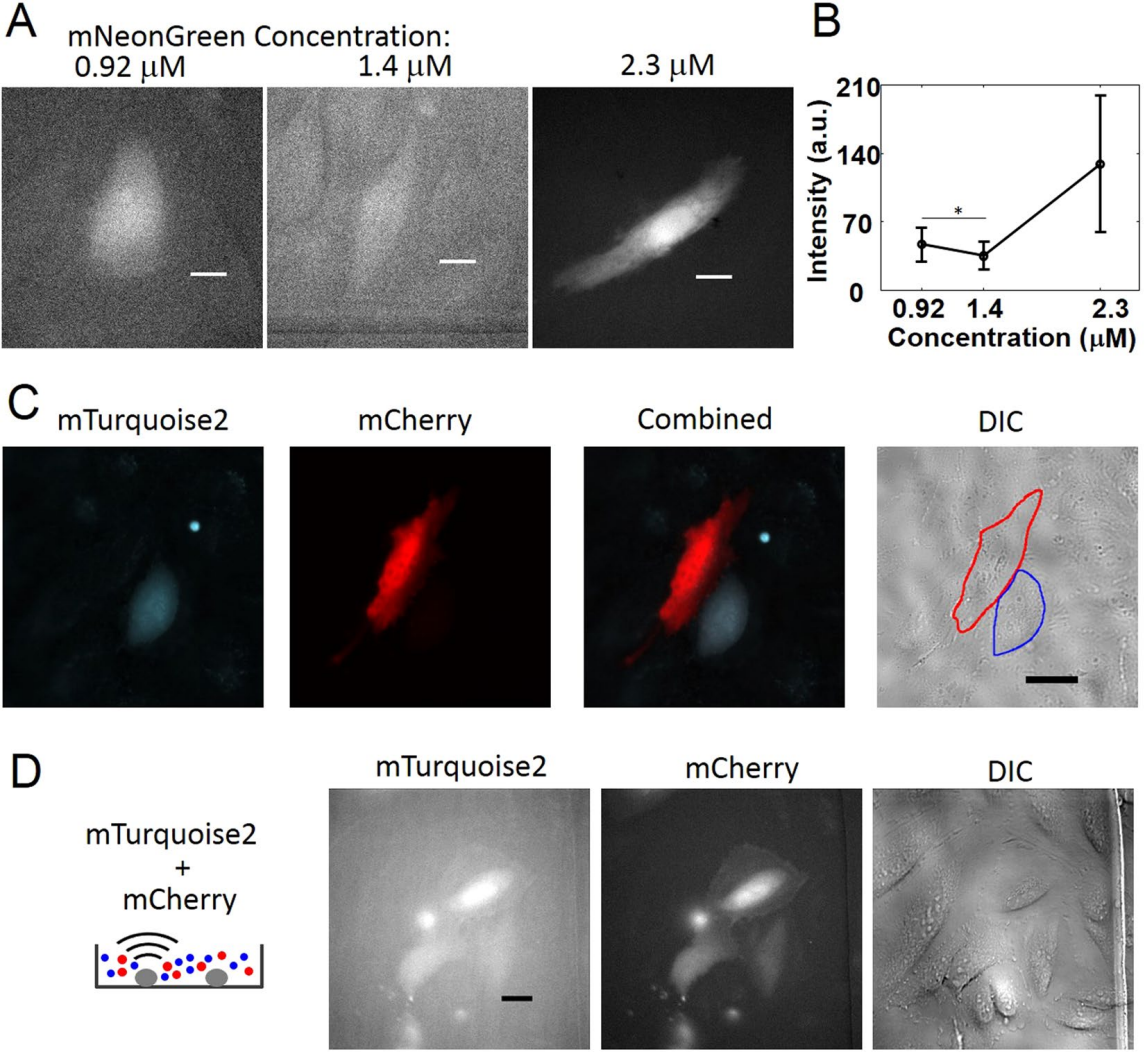
Protein delivery. The *Vp* and *tw* of each electrical pulse were 22 V and 18 μs, respectively, and the NP = 1. All images were taken 30 minutes after acoustic-transfection. (**A**) Representative images of the mNeonGreen (mNG) protein delivery under the mNG protein concentrations of 0.92 μM, 1.4 μM, and 2.3 μM are shown. (**B**) Six cells were used to calculate the fluorescence intensity of the mNG at each data point. Error bars represent +/− one SD. Asterisk (*) indicates that fluorescence intensities between 0.92 μM and 1.4 μM are not statistically significant (*p value* = 0.170). (**C**) The sequential delivery of mCherry (mCR) and mTurquoise2 (mTQ2) recombinant proteins into two neighboring target cells was confirmed. (**D**) Simultaneous delivery of mTQ2 and mCR into three neighboring HeLa cells was successfully performed. This shows a potential for a direct delivery of multiple reprogramming proteins for iPSC generation. All proteins have a molecular weight of approximately 27 kDa. Scale bars indicate 20 μm.

We purified His-tag fusion protein using pRSETB-mNeonGreen, mTurquoise2, and mCherry plasmids (Materials and Methods). Acoustic-transfected cells were imaged 30 minutes after the treatment using the GFP, CFP, or RFP channels. The concentrations of mNeonGreen protein were 0.92 μM, 1.4 μM, and 2.3 μM (Fig. 4A and B). The fluorescence intensity of the cells, transfected with mNeonGreen protein, was measured and plotted depending on the concentrations as shown in Fig. 4B. Increased protein concentration outside of the cell membrane induced increased intracellular delivery of mNeonGreen protein (Fig. 4B). We further acoustic-transfected mCherry and mTurquoise2 proteins into multiple cells sequentially (Fig. 4C) as well as simultaneously into adjacent cells with the protein concentration of 2.3 μM (Fig. 4D). The transfected cells were observed to be functioning normally up to 8 hours (Supplementary Video 2), suggesting the minimal cytotoxicity of our protein delivery technology.

### Gene knockin by CRISPR-Cas9 and acoustic-transfection

We demonstrated simultaneous delivery of three DNA plasmids into target cells using acoustic-transfection for the genome editing of target cells. Guide RNA (gRNA)^21^, hCas9 endonuclease^3^, and two types of donor repair templates sharing identical homology arms were all delivered into HeLa cells using acoustic-transfection and lipofectamine 3000 as a control. AAV-CAGGS-EGFP^4^ served as a control donor repair template to examine homologous-directed repair (HDR) by the CRISPR-Cas9 system. We cloned the second donor repair template to target F-actin using LifeAct^22^ with fluorescence marker TagRFP (AAV-LifeAct-TagRFP, details in Materials and Methods).

**Figure 5:**
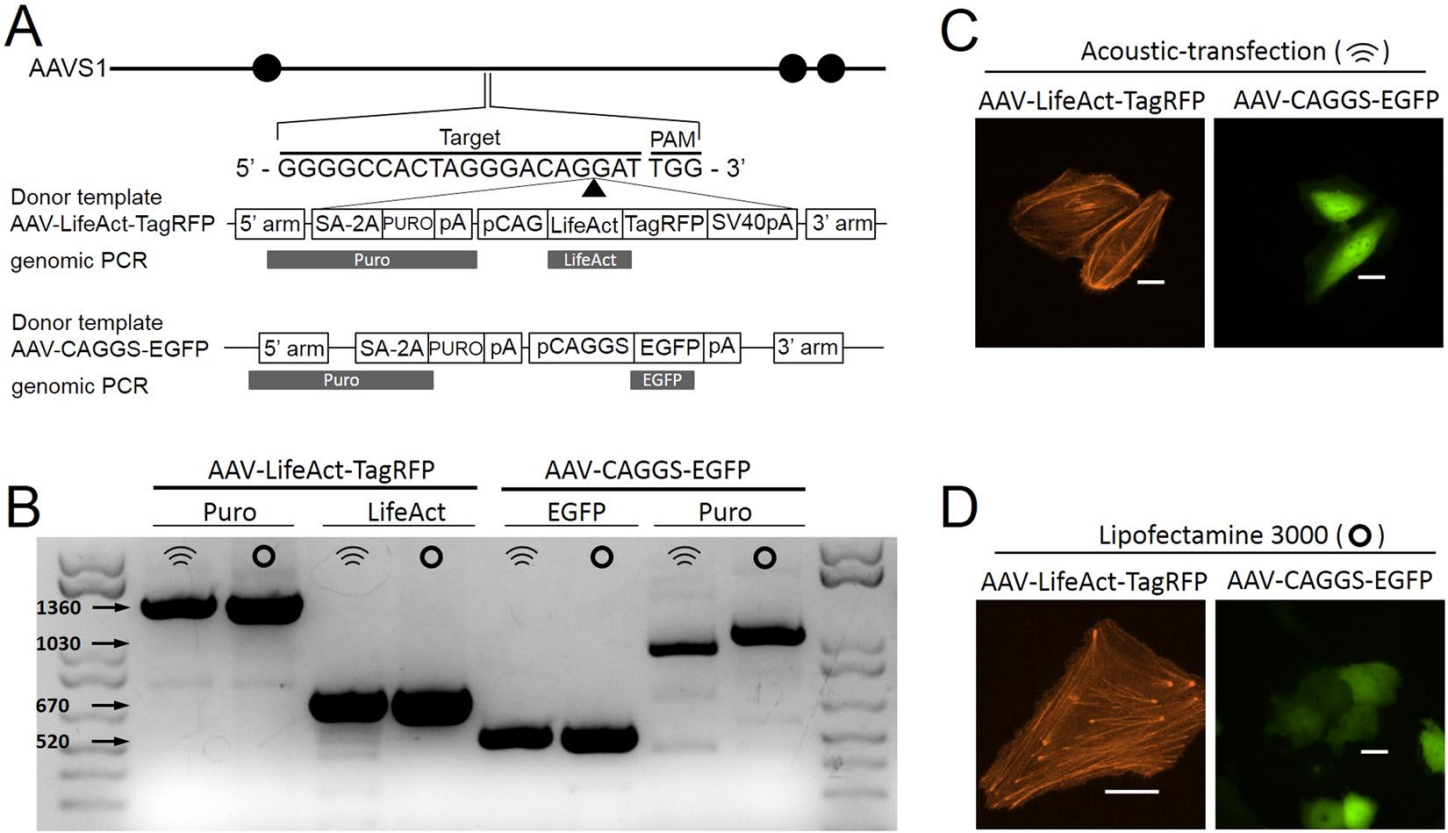
Gene knockin using acoustic-transfection and CRISPR-Cas9. (**A**) Schematic diagram of the targeting for the PPP1R12C gene in the AAVS1 locus. The three solid circles indicate first 3 exons of PPP1R12C. The dark grey boxes with Puro, LifeAct, and EGFP indicate the genomic PCR regions, thereby confirming the correct HDR events by CRISPR-Cas9, where the results are shown in (**B**). A solid arrow head represents the genomic cut by the CRISPR-Cas9. The 20-bp gRNA target and PAM sequences are also shown. Two types of donor repair templates for CRISPR-Cas9 targeting the locus are shown. SA-2A and PURO is the splice acceptor sequence followed by a 2A self-cleaving peptide sequence and the puromycin resistance gene. The pA is the polyadenylation sequence. The pCAG-LifeAct-TagRFP-SV40pA sequence is used to target the F-actin with a fluorescence marker (TagRFP) and pCAGGS-EGFP-pA is used to target the cytoplasm with EGFP. We cloned AAV-LifeAct-TagRFP and used AAV-CAGGS-EGFP from Addgene (#22212) as a control. To develop the AAV-LifeAct-TagRFP donor repair template, the pCAGGS-EGFP-pA sequence from AAV-CAGGS-EGFP was replaced with the pCAG-LifeAct-TagRFP-SV40pA sequence. The same gRNA and hCas9 were used for both CRISPR-Cas9 systems. (**B**) Arrows on the left indicate the expected positions of the DNA bands by genomic PCR. (**C**) Acoustic-transfection and (**D**) lipofectamine 3000 (as a control) were used to deliver two types of CRISPR-Cas9 systems. Representative images of the HeLa cells with gene expressions after the HDR by AAV-LifeAct-TagRFP (first panel) and AAV-CAGGS-EGFP (second panel) donor templates are presented in (**C**) and (**D**). Scale bars indicate 20 μm.

Gene editing was observed by HDR of fragments of AAV-CAGGS-EGFP and AAV-LifeAct-TagRFP into the endogenous PPP1R12C gene in the AAVS1 locus on chromosome 19 (Fig. 5). The gRNA target and PAM sequences in the PPP1R12C gene are shown in Fig. 5A. Fluorescence images of gene expression of the AAV-LifeAct-TagRFP and AAV-CAGGS-EGFP confirmed the delivery of the CRISPR-Cas9 system by acoustic-transfection (Fig. 5C) and lipofectamine 3000 (Fig. 5D). To verify the HDR gene editing, genomic PCR was conducted using appropriate primers (Supplementary Table 1C and D)^23^. Electrophoresis results show a correct match between the genomic PCR of the engineered HeLa cell genome by two CRISPR-Cas9 systems, delivered by acoustic-transfection and lipofectamine 3000 (Fig. 5B and Materials and Methods). The arrows in Fig. 5B indicate the expected positions of the bands from genomic PCR for the Puro, LifeAct, and EGFP sequences, as represented by the dark grey rectangles in Fig. 5A.

## Discussion

In this paper, we have described a high frequency ultrasound-based intracellular delivery technique termed acoustic-transfection. Ultrasound allows for noninvasive and remote manipulation at the single-cell level and deep tissue or organ level depending on the center frequency^8, 9, 24, 25^. High frequency ultrasound, with a center frequency of over 150 MHz, focuses acoustic energy into a very confined area with a diameter of 10 μm or less, which brings a single-cell level targeting into acoustic-transfection. The use of high frequency ultrasound addresses problems related to microbubble cavitation and non-specific targeting, which are issues of low frequency ultrasound-based sonoporation.

We also demonstrated that biologically active molecules can be delivered precisely by our method to allow the visualization and genome editing of the target cells. Macromolecules that can be delivered via acoustic-transfection are not restricted to nucleic acids. Recombinant proteins were successfully delivered into single-cells simultaneously and sequentially (Fig. 4). This technique, therefore, provides an opportunity for the generation of iPSCs by the delivery of proteins, capable of triggering reprogramming without the need for gene integration into the endogenous genome (as with viral-vector transduction)^6^. The ability to perform repeated delivery of diverse macromolecules into the same single-cells and longitudinal monitoring with high spatiotemporal resolution are additional advantages of acoustic-transfection when used for reprogramming of somatic cells. Furthermore, using acoustic-transfection with the CRISPR-Cas9 system should enable new strategies in gene editing^26, 27^. We demonstrated that gRNA, Cas9 nuclease, and two types of donor repair templates were successfully delivered into single-cells. In addition, successful insertion into the AAVS1 locus by HDR was confirmed (Fig. 5). As such, ultrasound can be applied to deliver genome editing reagents and remotely and noninvasively control the genome and functions of the target cells at single cell levels.

Physical methods for intracellular delivery of macromolecules such as electroporation, sonoporation, and microfluidics with constriction are based on a similar delivery mechanism^17–19^. Diffusion drives the transport of molecules through transient holes on the cell membrane^18^. We hypothesized that acoustic-transfection has a similar delivery mechanism. Because the delivery of molecules into the cell cytoplasm has been proven, we tested efflux of molecules through cell membrane with an increased permeability to confirm this hypothesis. We applied an acoustic pulse to one of the calcein loaded HeLa cells and a decrease of fluorescence intensity was clearly observed (Supplementary Video 3; Materials and Methods). Another issue for acoustic-transfection is the size of molecules that can be delivered because DNA plasmids, mRNAs, and proteins are large and hydrophilic. These characteristics make it difficult for them to spontaneously diffuse across cell membranes. We successfully tested the intracellular delivery of 70 kDa dextran labeled with OrangeGreen using acoustic-transfection (Supplementary Fig. 7). Even though the upper limit of the size of molecules was not identified, we concluded that molecules larger than 10 nm could be delivered by acoustic-transfection because the hydrodynamic diameter of 70 kDa dextran was measured to be approximately 14 nm^28, 29^. Because 70 kDa dextran is too large to pass through a nucleus envelope, the OrangeGreen signal (496(EX)/524(EM)) within a nucleus region is very weak as shown in Supplementary Fig. 7. To increase delivery efficiency of the DNA plasmids, mRNA, and proteins, chemical modifications or packaging using nanoparticles or lipids may be required in the future. In addition, packaging of the mRNA and recombinant proteins will prevent these molecules from degradation.

## Materials and Methods

### Input parameters for acoustic-transfection with low cytotoxicity

Single-cell targeting and the increase of membrane permeability for noninvasive and remote molecular transport are the most important functions of a high frequency ultrasonic transducer for acoustic-transfection. The lateral beam profile, *δ*_*L*_, (Equation S3 in Supplementary Note 3 and Supplementary Fig. 3) of an acoustic beam must be narrow enough to target single-cells. The focusing gain, *G*, (Equation S4 in Supplementary Note 3 and Supplementary Fig. 3) of the acoustic beam should be at a sufficiently high level to disturb the cell membrane in order to increase membrane permeability within the safe regime. While low frequency ultrasonic transducers typically have a wide *δ*_*L*_ (0.5–2 mm) and low *G* (1–5), a narrow *δ*_*L*_ of 10 μm and a high *G* of 79 were achieved with our unique high frequency ultrasonic transducer design (Supplementary Note 3 and Supplementary Fig. 3). We built a high frequency ultrasonic transducer according to the design and the fabrication protocol developed by our laboratory as described below in the Materials and Methods section. Axial and lateral resolutions were experimentally measured to be 8.5 μm and 10 μm, respectively, thereby ensuring the confinement of the acoustic energy to a single-cell level area (Supplementary Note 1 and Supplementary Fig. 4). The acoustic-transfection system setup and its operation are also explained in the Materials and Methods section.

According to our previous optimization study of input parameters (Fig. 1C2), we chose peak-to-peak voltage (*Vp*) of 22 V, pulse width (*tw*) of 18 μs, and the number of pulses (NP) of 1 throughout the experiments in this paper to perform acoustic-transfection with low cytotoxicity^20^. An indirect cell viability test utilizing a fluorescent marker (calcein-AM) was performed to indicate the integrity of the cell plasma membrane (details in Materials and Methods). The results indicated 100% cell viability 6 hours after acoustic-transfection when *Vp = *22 V, *tw* = 23 μs, and NP = 1 (Supplementary Fig. 5).

### Construction of AAV-LifeAct-TagRFP donor repair template for CRISPR-Cas9

AAV-CAGGS-EGFP (Addgene plasmid #22212) was PCR amplified using Q5 High-Fidelity DNA Polymerase (New England Biolabs, Ipswich, Massachusetts) and FWD and rev primers (Supplementary Table 1A). p^CAG^ LifeAct-TagRFP (ibidi GmbH, Germany)^22^ was digested using CutSmart (New England Biolabs, Ipswich, Massachusetts) at AflII and SalI sites. The PCR product of AAV-CAGGS-EGFP and digested p^CAG^ LifeAct-TagRFP were ligated using T4 DNA Ligase (New England Biolabs, Ipswich, Massachusetts) to construct AAV-LifeAct-TagRFP donor repair template.

### DNA plasmids preparation

Four DNA plasmids (pMax-EGFP (3.5 kb); FRET-BS-CyanYPET (6.7 kb); pCas9-EGFP (9.3 kb); Histone-H3-targeted-YPET (10.2 kb)) for delivery efficiency experiments in Fig. 2, four DNA plasmids (gRNA (Addgene plasmid #47108, 3.2 kb); hCas9 (Addgene plasmid #41815, 9.6 kb); AAV-CAGGS-EGFP; AAV-LifeAct-TagRFP) for CRISPR-Cas9 experiments in Fig. 5, and FRET- BS-GreenRed in Fig. 3 were prepared using the HiSpeed Plasmid Maxi Kit (Cat #. 12663, Qiagen, Germany). The concentrations of these DNA plasmids were measured to be between 600 ng/μl and 1400 ng/μl using a NanoDrop 2000 UV-Vis Spectrophotometer (Thermo Fisher Scientific, Waltham, Massachusetts).

### Protein purification and mRNA generation

mNeonGreen, mTurquoise2, and mCherry fluorescent protein coding sequences were inserted between BamHI and EcoRI sites in pRSETB (Thermo Fisher Scientific, Waltham, Massachusetts) with a 6XHis tag. Sequences for all primers are listed in Supplementary Table 1B. Proteins were expressed in *E*.*coli* BL21 (Promega, Madison, Wisconsin) competent cells under the control of a T7 promoter overnight in 37 °C LB medium with ampicillin at 250 r.p.m, followed by IPTG induction. B-Per II Bacterial Protein Extraction Reagent (Thermo Fisher Scientific, Waltham, Massachusetts) was used to lyse bacteria in order to extract protein. Ni-NTA affinity resin (Qiagen, Germany) was used to purify His-Tagged proteins by gravity flow chromatography for approximately 1 hour. We washed and eluted purified proteins using a wash buffer (50 mM Tris, pH = 7.4, 300 mM NaCl, 10 mM imidazole) and an elution buffer (50 mM Tris, pH = 7.4, 300 mM NaCl, 100 mM imidazole). The protein solution was dialyzed overnight in a dialysis buffer (20 mM HEPES, pH = 7.4, 500 mM NaCl) using Snakeskin™ dialysis tubing (Thermo Fisher Scientific, Waltham, Massachusetts). The concentrations of mNeonGreen, mTurquoise2, and mCherry proteins were measured using NanoDrop 2000 UV-Vis Spectrophotometer with an extinction coefficient of 116, 30, 72 (mM^−1^ . cm^−1^)^30–32^. The protein was stored in a dialysis buffer at −80 °C in a freezer after aliquot.

The mRNA strands were generated by *in vitro* transcription using linearized pRSETB-mNeonGreen, mTurquoise2, and mCherry vectors with a T7 mScript™ Standard mRNA Production System (CellScript, Madison, Wisconsin), according to the manufacturer’s instructions. The concentration of each mRNA was measured using a NanoDrop 2000 UV-Vis Spectrophotometer. The mRNA strands were aliquoted and stored at −80 °C in a freezer. Before acoustic-transfection experiments, each mRNA strand was diluted with HBSS with Ca^2+^ (Thermo Fisher Scientific, Waltham, Massachusetts) to a concentration of 0.2 μg/μl and transferred to petri dishes for acoustic-transfection.

### High frequency ultrasonic transducer fabrication

The transducer was developed in the Ultrasonic Transducer Research Center (Director: K. Kirk Shung) at the University of Southern California. The acoustic stack was composed of a backing layer (BL in Fig. 1A) and lithium niobate (LN in Fig. 1A). The LN generates acoustic pulses for acoustic-transfection by the excitation of an electrical signal (Fig. 1C). A 36° rotated Y-cut lithium niobate plate (Boston Piezo-Optics, Bellingham, Massachusetts) was lapped down to the desired thickness of 10 μm. A conductive silver epoxy (E-Solder 3022, Von Roll Isola Inc., New Haven, Connecticut) was cast on one side of the LN to form a BL with a thickness of approximately 1 mm. The acoustic stack was machined to be a cylindrical shape with an aperture of 1 mm (*ap* in Fig. 1A) using a lathe. The acoustic stack was press-focused with a stainless steel ball of 2 mm diameter to create a focal distance at 1.0 mm (*d* in Fig. 1A). The *fnumber* of the acoustic stack was 1.0. At the distal end of a stainless steel hypodermic needle (housing in Fig. 1A), the acoustic stack was inserted and fixed by insulating epoxy (IE in Fig. 1A, Epo-Tek 301, Epoxy Technologies, Billerica, Massachusetts). A silver wire with a thin insulating jacket (hot wire in Fig. 1A) was connected to the BL of the acoustic stack using a conductive silver epoxy for electrical connection.

The entire area of the distal end of the stainless steel needle (housing), including the acoustic stack, was sputtered with a chrome/gold electrode at a thickness of approximately 150 nm. This ensured a ground connection between the acoustic stacks and the housing. The silver wire (hot wire) from the acoustic stack was connected to a connector. As the last step of the fabrication process, parylene was deposited on top of the acoustic stack using a PDS 2010 Labcoater (Specialty Coating Systems, Indianapolis, Indiana). The thickness of the parylene coating was 1.0 μm to protect the transducer from corrosion in water.

### Acoustic-transfection system setup

A 3D axis stage (SGSP 20, Sigma KOKI Co., Japan) was integrated with a Nikon epifluorescence microscope (Eclipse Ti-U, Melville, New York) to perform live cell imaging during and after the acoustic-transfection (Fig. 1B). The developed high frequency ultrasonic transducer was mounted on the 3D axis stage, controlled by a stage controller (SHOT 202, Sigma KOKI Co., Japan) to accurately control the location of the ultrasonic transducer (Fig. 1B).

### Cell culture and cell preparation for acoustic-transfection

Human cervical carcinoma cells (HeLa, ATCC, Manassas, Virginia) and Human Embryonic Kidney cells (HEK293, ATCC, Manassas, Virginia) were cultured in DMEM with a 10% FBS at 37 °C and 5% CO_2_ in a humidified incubator. Cultures were passaged every 2–4 days depending on confluency using 0.025% trypsin-EDTA and PBS.

For the acoustic-transfection experiments, 50,000–100,000 cells were seeded on µ-Dish-Grid (ibidi GmbH, Germany) and incubated for 12 hrs–18 hrs under normal cell culture conditions before acoustic-transfection. To perform acoustic-transfection, HBSS with Ca^2+^ was mixed with target molecules to the designated concentration and placed in the µ-Dish-Grid (Fig. 1C). After acoustic-transfection, we relocated the same cells under epifluorescence microscope for imaging analysis using the grid on the dish. To check acoustic-transfection efficiency and fluorescence intensity of gene expression, the cell culture medium was changed to HBSS with Ca^2+^ for clear images.

### Operation of acoustic-transfection system

The ultrasonic transducer was connected to a pulser/receiver (DPR500 pulser/receiver, Imaginant Inc. Pittsford, New York) and an oscilloscope (display) (Fig. 1C1. focusing mode). While the ultrasonic transducer was moved vertically under the control of a stage controller, the maximum echo signal at the oscilloscope (display) indicated the focus of the ultrasonic transducer was correctly placed on the bottom surface of the petridish. Then, the ultrasonic transducer was moved horizontally to align the foci of the ultrasonic transducer and the objective lens, as shown by the solid box in Fig. 1C1. A target cell was co-aligned with the ultrasonic transducer and the objective lens of microscope.

The ultrasonic transducer was, then, connected to a power amplifier (525LA, ENI, Rochester, New York) with a 50 dB gain and a function generator (33250 A, Keysight Technologies, Santa Rosa, California) to generate the acoustic pulse required for acoustic-transfection (Fig. 1C2 acoustic pulse generation mode). A function generator emitted an electrical signal, which was amplified by a power amplifier to excite the ultrasonic transducer. The input parameters, including *Vp*, *tw*, PRT, and NP were accurately controlled by a function generator. As shown by the red solid box in Fig. 1C2, the generated acoustic pulse was used to acoustic-transfect a target cell to deliver macromolecules.

For CFP and mTurquoise2 fluorescence imaging, we used 420/40 nm excitation and 480/40 nm emission filters with a 455 nm dichroic mirror. For mCherry and RFP fluorescence imaging, 560/40 nm excitation and 650/100 nm emission filters with a 585 nm dichroic mirror were used. For GFP and mNeonGreen fluorescence imaging, 470/40 nm excitation and 525/50 nm emission filters with a 495 nm dichroic mirror were used. For TagRFP fluorescence imaging, 560/40 nm excitation and 650/100 nm emission filters with a 560 nm dichroic mirror were used.

### Data analysis

Fluorescence intensity of gene and protein expression in Figs 2E,G and 4B were measured offline using ImageJ software. The region of interest (ROI) was manually defined by covering the entire cytoplasm of a target cell using the ImageJ ROI tool. The average fluorescence intensity was automatically calculated by ImageJ.

### Lipofectamine 3000 transfection of CRISPR-Cas9

Lipofectamine 3000 (Thermo Fisher Scientific, Waltham, Massachusetts) was used to transfect two CRISPR-Cas9 systems to target cytoplasm with EGFP and F-actin with TagRFP into HeLa cells, following the manufacturer’s instructions. 333 ng of RNA, Cas9 nuclease, and donor templates were added into cell growth medium in 35 mm dishes.

### Genomic PCR

We designed primers for the genomic PCR using primer-blast and all primers that were used for genomic PCR are listed in Supplementary Table 1C and D. We performed puromycin selection for 5 days starting 60 hours after the initial delivery of CRISPR-Cas9 by acoustic-transfection and lipofectamine 3000. We expanded the cells for 5 days after the puromycin selection and then harvested the cells to extract the genomic DNA using a DNeasy Blood & Tissue Kit (Qiagen, Germany). We designed the primers using primer-blasts^23^ to amplify a part of the 5′ homology right arm and puromycin sequences with different lengths (1030 nt and 1360 nt) as well as LifeAct (670 nt) and EGFP (520 nt) sequences with the genomic DNA derived from the cells by genomic PCR (Fig. 5A and B and Supplementary Table 1).

### 70 kDa dextran and calcein-AM

70 kDa dextran labeled with OrangeGreen and calcein-AM were purchased from Thermo Fisher Scientific (Waltham, Massachusetts). The 70 kDa dextran was diluted into HBSS with Ca^2+^ and the concentration was 71 μM for acoustic-transfection.

Six hours after acoustic-transfection, the HBSS with Ca^2+^ and calcein-AM were mixed and incubated with the treated cells in a petridish for approximately 20 minutes. The cells with an intact cell membrane showed green fluorescence (488 (EX)/520 (EM) nm), which can be considered as an indirect indicator of cell viability. Cell viability was calculated the number of cells with green fluorescence out of total number of acoustic-transfected cells (n = 18, a representative image in Supplementary Fig. 5).

To observe efflux of molecules from cytoplasm of cells, HeLa cells were loaded with calcein-AM according to manufacturer’s instructions. Acoustic-transfection was applied to one single-cell. Every 0.5 second, the fluorescence images of the calcein loaded HeLa cells were saved. Saved images were stacked and converted to a movie file as shown in Supplementary Video 3.

## Electronic supplementary material

Supplementary information

Supplementary Video 1

Supplementary Video 2

Supplementary Video 3
