# Supplementary material for: Acoustic-transfection for genomic manipulation of single-cells using high frequency ultrasound
Source: Sci Rep. 2017 Jul 13;7:5275. doi: 10.1038/s41598-017-05722-1 (PMC5509725; doi:10.1038/s41598-017-05722-1)
Supplement: Supplementary file 1 — Supplementary information [file 41598_2017_5722_MOESM1_ESM.pdf]

Acoustic-transfection for genomic manipulation of single-cells using high frequency ultrasound

Sangpil Yoon[1], Pengzhi Wang[2], Qin Peng[2], Yingxiao Wang[2,*], K. Kirk Shung[1,*]

[1]Department of Biomedical Engineering, University of Southern California, 1042 Downey Way, Los Angeles, California 90089, USA

[2]Department of Bioengineering & Institute of Engineering in Medicine, University of California, San Diego, 9500 Gilman Drive, La Jolla, California 92093, USA

* Corresponding Authors:
Yingxiao Wang, Ph.D., 9500 Gilman Drive, SERF 287, La Jolla CA, 92093;
yiw015@eng.ucsd.edu; +1-858-822-4502

K. Kirk Shung, Ph.D., 1042 Downey Way, DRB-136, Los Angeles CA, 90089;
kkshung@usc.edu; +1-213-821-2653

Supplementary Note 1. Estimation of pulse / echo and spatial resolution of a high frequency ultrasonic transducer. Pulse / echo time and frequency responses were measured to find a center frequency (*f*) of a high frequency ultrasonic transducer using a custom-made ultrasound biomicroscopy (UBM) system. The schematic diagram of the UBM is shown in Supplementary Fig. 1. The UBM system was composed of a main computer, a pulser / receiver (DPR500 pulser / receiver, Imaginant Inc. Pittsford, New York), a 3D axis stage (ILS100HA, Newport, Irvine, California) with a stage controller (ESP301-3N, Newport, Irvine, California), and an analog-to-digital acquisition card (A/D card, CS122G1, DynamicsSignals LLC., Lockport, Illinois). A quartz block was used as a reflecting target for the pulse / echo measurements at the focus of the high frequency ultrasonic transducer. The high frequency ultrasonic transducer was mounted on a 3D axis stage. Pulses from a pulser / receiver excited the high frequency ultrasonic transducer with 200 Hz pulse repetition frequency (PRF). The reflected echoes were received by the same ultrasonic transducer and amplified by the same pulser / receiver (Supplementary Fig. 1). The pulses and amplified echoes were saved and post-processed by a custom-built Matlab (The MathWorks Inc., Natick, Massachusetts) program to measure time domain echo waveforms and their frequency spectra, as shown in Supplementary Fig. 1C. Real measurements of the time domain echo waveforms and frequency spectrum of the ultrasound transducer are shown in Supplementary Figs. 4A and 4B. The center frequency (*f*) was determined by

$$f = \frac{Cut\text{-}off1 + Cut\text{-}off2}{2}, \qquad (S1)$$

where cut-off1 and cut-off2 are the -6 dB frequencies from the estimated spectra as shown in Supplementary Fig. 4B.

For the axial and lateral resolutions measurements, the high frequency ultrasonic transducer was translated with a step size of 1 μm to acquire the scan lines required to obtain 2D cross-sectional images of a tungsten wire target, as shown in Supplementary Fig. 1. A tungsten wire target with a diameter of 4 μm was used for resolution characterization. The point spread function (PSF) at

the focus of the ultrasonic transducer was plotted along the axial and lateral directions from the 2D cross-sectional images of the tungsten wire target to measure the axial and lateral (δ_L) resolutions, as shown in Supplementary Figs. 4C and 4E. The axial and lateral resolutions were then determined by using the −6 dB points on the PSF plots.

Supplementary Note 2. Ultrasonic transducer design guideline. We established the following guideline for designing ultrasonic transducers for acoustic-transfection even though a complete optimization of every design parameter of the ultrasonic transducers was not performed. When designing an ultrasonic transducer, space constraints and the center frequency of the transducer need to be considered. Space constraints restrict the size of aperture (*ap*) and focal distance (*d*), which determines the f_{number} (Fig. 1A). A large aperture and f_{number} will occupy more spaces. Therefore, if acoustic-transfection and microfluidic channels are combined for intracellular delivery, a small aperture and f_{number} are desired. Once the fabrication of the ultrasonic transducers is completed, the strength of acoustic-transfection is easily controlled by changing the input parameters (V_p, t_w, PRT, and NP) of the electrical signal using an electronic control system such as a power amplifier and function generator (Fig. 1C2).

The level of the normalized pressure ($\bar{p}$, nondimensionalized) at the focus of an ultrasonic transducer is simulated in Supplementary Fig. 2. The angular spectrum method was used for simulation [1]. Normalized pressure is

$$\bar{p} = \frac{p}{\rho_0 c_0 u}, \qquad (S2)$$

where p is the real pressure at the focus, ρ_0 is the density of water, c_0 is the speed of sound, and u is the velocity of the aperture. An ultrasonic transducer with a larger *ap*, smaller f_{number}, and higher frequency generates a higher level of pressure at the focus, as shown in Supplementary Fig. 2. Under the same input parameters of an electrical signal (Fig. 1C2), the pressure level at the focus of an ultrasonic transducer can be deduced by using Supplementary Fig. 2. Usually,

pressure levels at the focus and the input parameters of an electrical signal exhibit a nonlinear relationship. A solid dot in Supplementary Fig. 2 indicates the normalized pressure at the focus of the ultrasonic transducer, used in this study.

Supplementary Note 3. Lateral resolution and focusing gain. The governing equations to define lateral beam profile (δ_L) and focusing gain (G) are

$$\delta_L = f_{number}\lambda = f_{number}\frac{c}{f} \quad , \qquad (S3)$$

$$G = \frac{S}{d \cdot \lambda} = \frac{S}{d \cdot {}^{c}/_{f}} \quad , \qquad (S4)$$

where *f*$_{number}$ is the ratio of focal distance to the aperture as shown in Fig. 1A, λ is the wavelength of the acoustic sound waves, c is the speed of sound in water (1.5 mm/μs), *f* is the center frequency of the acoustic sound waves (Equation S1 in Supplementary Note 1), *S* is the area of the aperture, defined as $\pi(ap)^2$, and *d* is the focal distance (Fig. 1A). In this study, we designed *ap*, *d*, and *f* of the ultrasonic transducer to be 1 mm, 1 mm, and 150 MHz, respectively, resulting in values for δ_L and G to be 10 μm and 79, respectively.

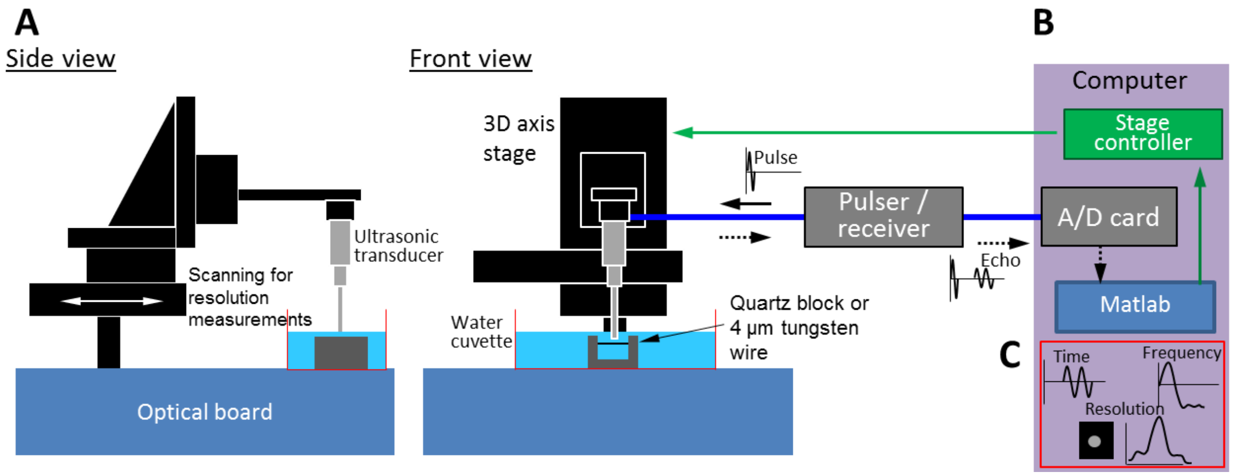

Supplementary Figure 1. A schematic diagram of ultrasound biomicroscopy (UBM) system for pulse / echo and spatial resolution measurements. (A) A 3D axis stage and a water cuvette were placed on an optical board. The 3D axis stage precisely changed the location of the high frequency ultrasonic transducer (grey color). A quartz block was placed in a water cuvette at the focus of the ultrasonic transducer to measure a center frequency of the transducer. The transducer was connected to a pulser / receiver and an A/D card. A stage controller manipulated the 3D axis stage. (B) Both the stage controller and A/D card were controlled by custom-built Matlab program to acquire the pulse / echo wave form. The pulse and reflected echo signals, reflected by a quartz block, were saved and stored for offline processing. (C) A custom-built Matlab program was used to generate the frequency spectrum of the echo signal to determine a center frequency of the ultrasonic transducer (see Supplementary Fig. 4). For spatial resolution measurements, a 4 µm tungsten wire was used. (B) By moving the ultrasonic transducer with at increments of 1 µm, 100 scanlines, which encompass a 100 µm distance including a 4 µm tungsten wire, were generated and saved for offline processing. (C) 2D grey scale images of the tungsten wire was reconstructed via a custom-built Matlab program to generate point spread function (PSF). The axial and lateral resolutions were estimated (see Supplementary Fig. 4).

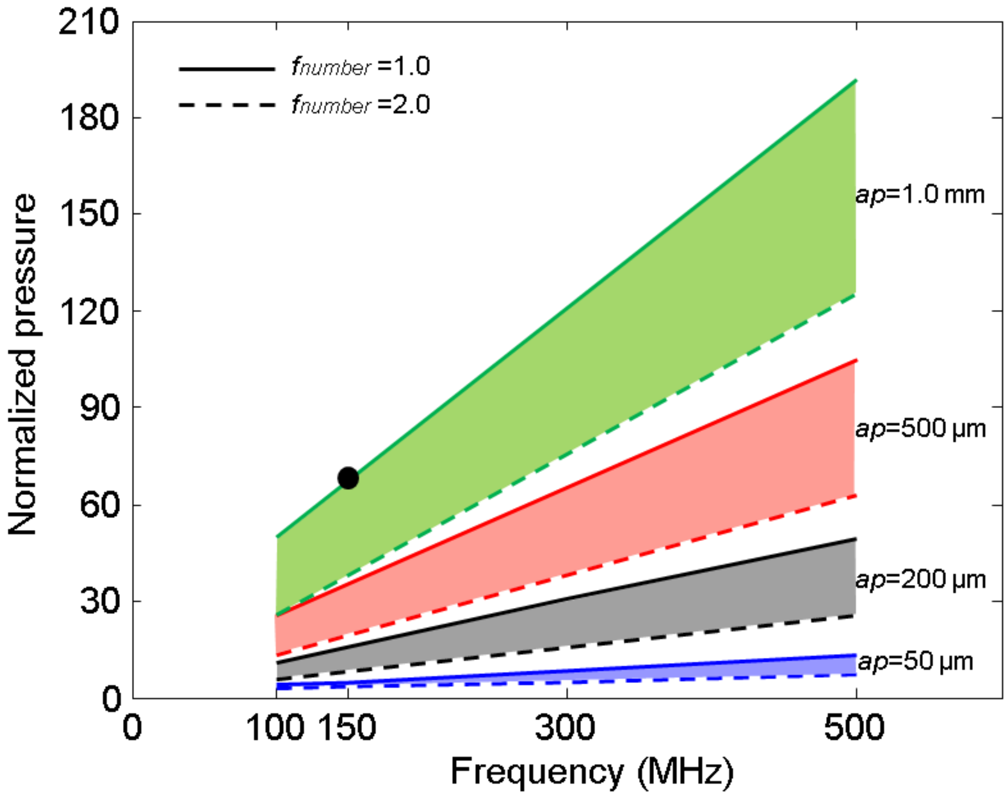

Supplementary Figure 2. Simulation results of the normalized pressure (Equation S2 in Supplementary Note 2) at the focus of the high frequency ultrasonic transducer depending on center frequency, aperture, and f_{number}. A higher pressure is generated at the focus of the transducer when a larger aperture, *ap* (Green area), smaller f_{number} (Solid line), and higher center frequency were utilized. The solid circle is the normalized pressure level of the ultrasonic transducer used.

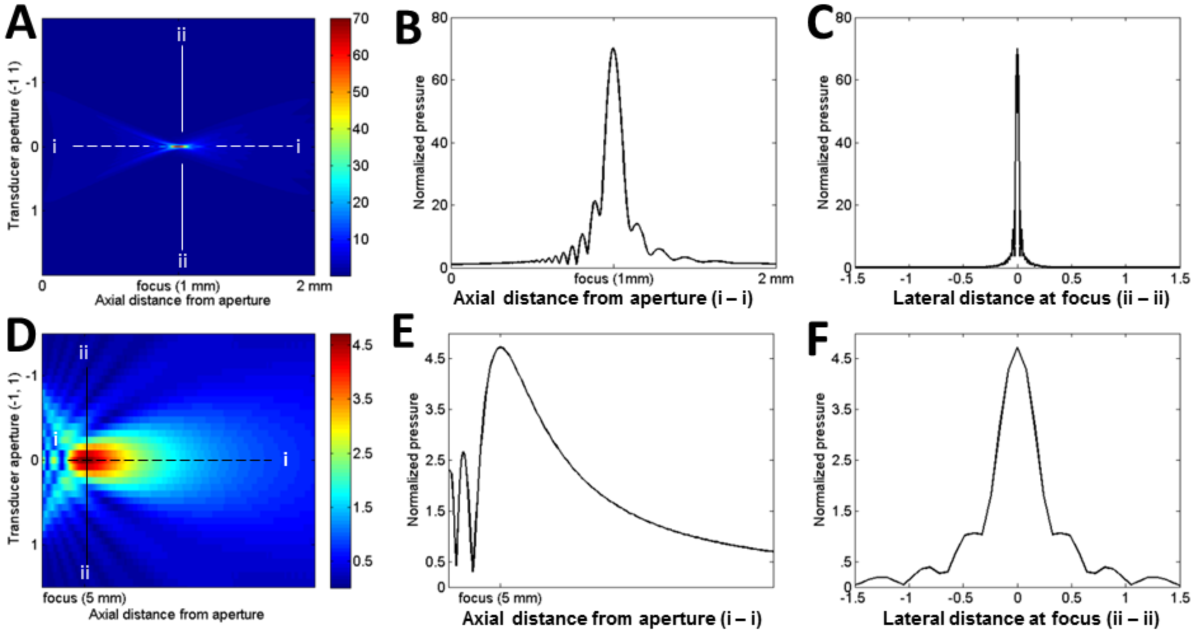

Supplementary Figure 3. Comparison of lateral resolution and focusing gain between high frequency and low frequency ultrasonic transducers. The first row indicates the simulation results of (A) 2D pressure field, (B) on-axis pressure (i–i), and (C) lateral pressure profile at focus (ii–ii) for a 150 MHz high frequency ultrasonic transducer. The second row indicates the simulation results of (D) 2D pressure field, (E) on-axis pressure (i–i), and (F) lateral pressure profile at focus (ii – ii) of a 2 MHz low frequency ultrasonic transducer. The foci of the ultrasonic transducers are located at 1 mm for 150 MHz and 5 mm for 2 MHz, as shown in x-axis at (A) and (D). Both transducers have the same f_{number} of 1. The aperture of the ultrasonic transducer is located between -1 and 1 on the y-axis at (A) and (D). The acoustic sound waves propagate from left to right in (A), (B), (D), and (E). The simulated pressure was normalized by the impedance and source velocity of the transducer aperture (Equation S1 in Supplementary Note 1). The normalized peak pressure of the 150 MHz ultrasound field is approximately 71 and simulated lateral resolution is approximately 10 μm. Alternatively, for the 2 MHz ultrasound pressure field, the normalized peak pressure is only 4.6 and lateral resolution is approximately 800 μm. Therefore, the 150 MHz ultrasonic transducer can effectively concentrate acoustic energy into a confined area when compared to the 2 MHz transducer.

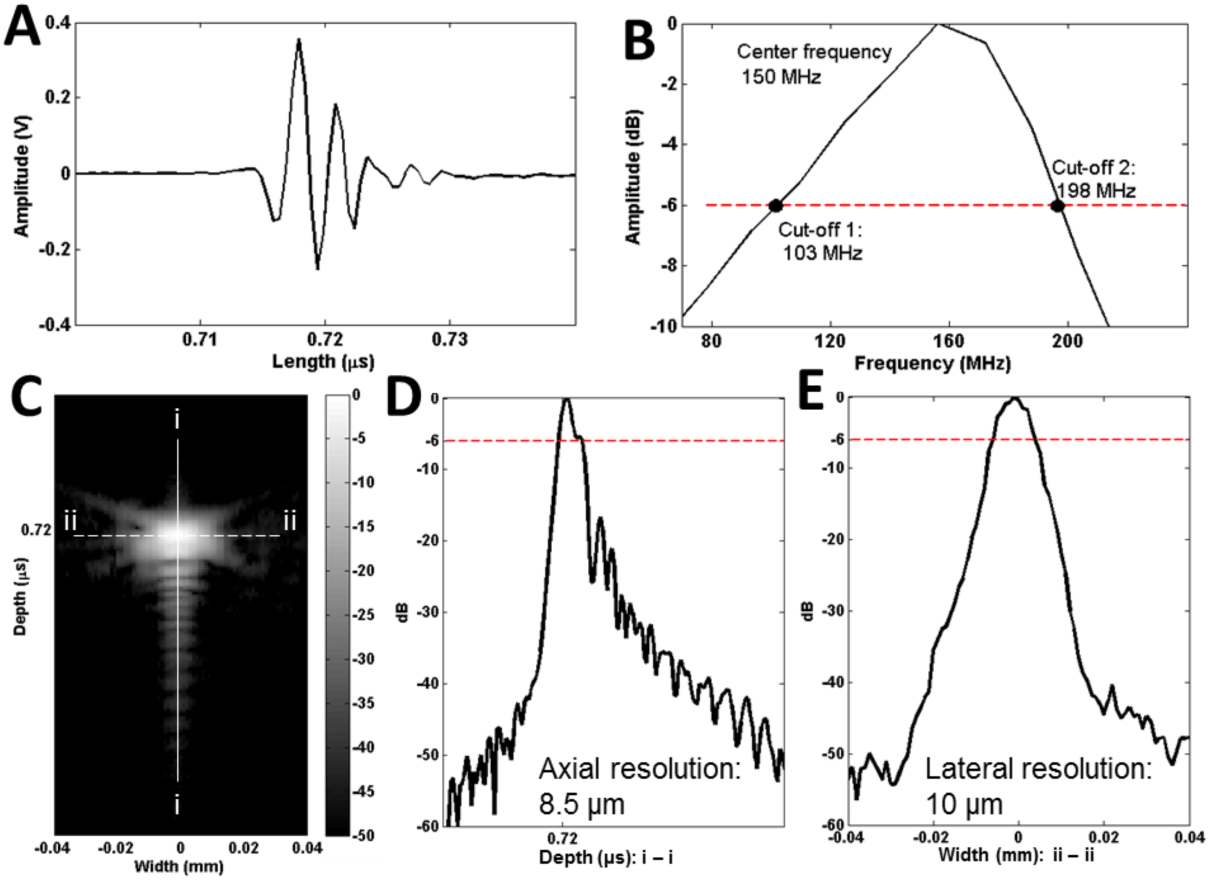

Supplementary Figure 4. High frequency ultrasonic transducer characterization to confirm single-cell level targeting of acoustic-transfection. (A) Time domain pulse-echo waveform and (B) frequency spectrum measurements indicate that focal distance (*d*) is located at 1.06 mm away from the transducer's aperture and the center frequency is measured to be 150 MHz. (C) The 2D gray scale intensity profile of 4 μm tungsten wire acquired by the high frequency ultrasonic transducer shows the uniform focusing capability of the fabricated high frequency ultrasonic transducer. (D) Axial and (E) lateral resolutions were measured to be 8.5 μm and 10 μm by taking the width of -6 dB points on the 2D gray scale intensity. The procedure for measuring the center frequency and spatial resolution measurements is described in Supplementary Note 1.

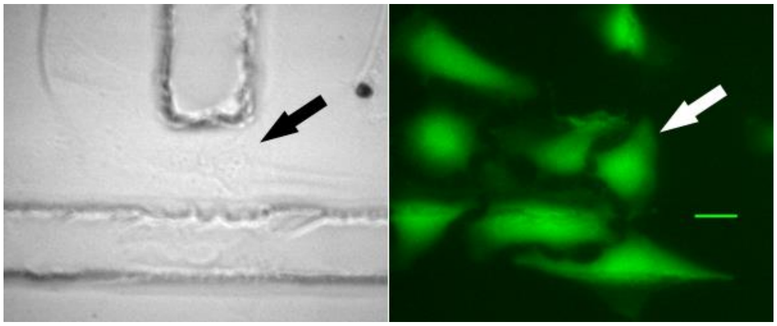

Supplementary Figure 5. Calcein-AM staining after acoustic-transfection with V_p of 22 V, t_w of 23 μs, and NP of 1. Calcein-AM staining was performed 6 hours after the acoustic-transfection. Representative DIC and fluorescence images of an acoustic-transfected HeLa cell is indicated by the arrows. The green fluorescence of the acoustic-transfected HeLa cell represents an intact cell plasma membrane, which is an indirect gauge of cell viability. All acoustic-transfected cells showed green fluorescence and we concluded that the condition for acoustic-transfection (V_p= 22 V; t_w= 18 μs; NP= 1) is within safe range with 100% cell survival rate (n=18). (Scale bar, 20 μm)

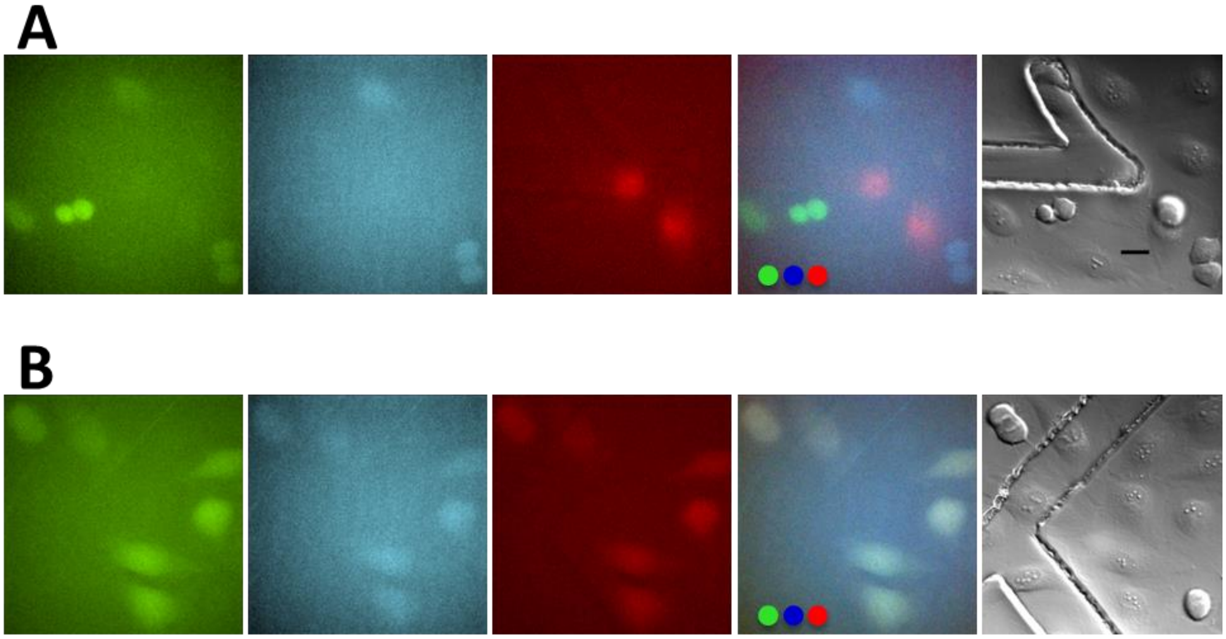

Supplementary Figure 6. Sequential single-cell targeting and simultaneous acoustic-transfection of mRNA (40 hours tracking). (A) The mRNA strands expressing mNeonGreen, mTurquoise2, and mCherry were sequentially transfected into neighboring cells and imaged 40 hours after the acoustic-transfection. The same cells as in Fig. 3B were studied again. (B) Simultaneous delivery of three types of mRNA strands into three cells by acoustic-transfection. Same cells as in Fig. 3C. (Scale bar, 20 μm)

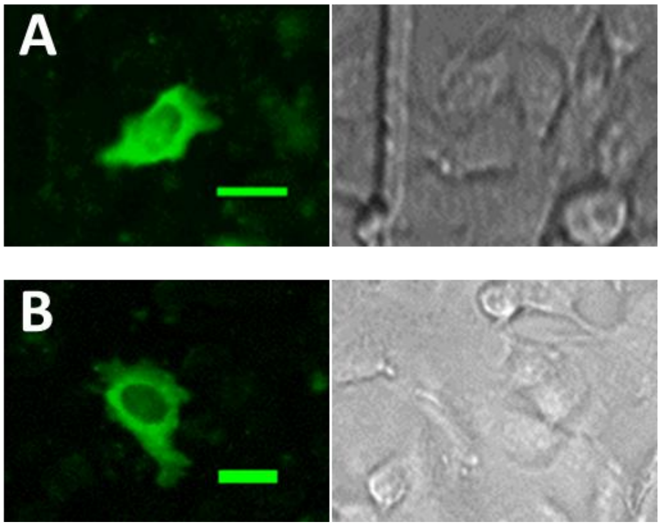

Supplementary Figure 7. Intracellular delivery of 70 kDa dextran labeled with OrangeGreen by acoustic-transfection. (A–B) HeLa cells were acoustic-transfected with 70 kDa dextran. The fluorescence and DIC images were taken 30 minutes after acoustic-transfection. The hydrodynamic diameter of 70 kDa dextran is approximately 14 nm. The nucleus region does not have a fluorescence signal because the 70 kDa dextran is too large to pass through a nucleus envelope. (Scale bar, 20 μm)

Supplementary Table 1. Primers for PCR used in this study

A. AAV-LifeAct-TagRFP donor repair template

FWD	aaatCTTAAGCGACAGTACTAAGCTTTACTAGGGA	underline AflII site
rev	aaatGTCGACCCATAGAGCCCACCGCATC	underline SalI site

B. pRSETB-(mNeonGreen, mTurquoise2, mCherry) vectors for protein purification

FWD	aaatggatccCGTCTCAATGGTGAGCAAGGGCGAGGA	underline BamHI
rev	aaatgaattccgtctcaTTACTTGTACAGCTCGTCCA	underline EcoRI

C. Genomic PCR to analyze the HDR events by CRISPR-Cas9 with AAV-LifeAct-TagRFP

Puro	FWD	AGCTGGGACCACCTTATATT
	rev	GTCAATAGGGGGCGTACTTG
LifeAct	FWD	ATGGGTGTCGCAGATTTGAT
	rev	GGTCCACATAGTAGACGCCG

D. Genomic PCR to analyze the HDR events by CRISPR-Cas9 with AAV-CAGGS-EGFP

Puro	FWD	CTGCCGTCTCTCTCCTGAGT
	rev	TAGGCTCGAGATGACCGAGT
EGFP	FWD	TTGGCAAAGAATTCCGCCAC
	rev	AGAACGGCATCAAGGTGAACT

Supplementary Video 1. The pCas9-EGFP plasmid expression after acoustic-transfection. Time-lapse imaging of the transient EGFP expression starting 3 hours after acoustic-transfection of the pCas9-EGFP plasmid into a HeLa cell. Images were taken every 15 minutes for 18.5 hours. Playback is at 7 frames per second. EGFP intensities were not modified.

Supplementary Video 2. The mNeonGreen protein delivery into a single-cell using acoustic-transfection. Time-lapse imaging of a mNeonGreen acoustic-transfected HeLa cell. Images were taken 1 hour after acoustic-transfection every 20 minutes for 7 hours. Playback is at 7 frames per second. The mNeonGreen intensities were not modified.

Supplementary Video 3. Calcein efflux due to diffusion after an acoustic pulse on a target cell. Time-lapse imaging of calein loaded HeLa cells on the left and a plot of calcein fluorescence intensity of a target cell on the right. After calcein was loaded on the cells, an acoustic pulse was applied to only one HeLa cell. Shortly after an acoustic pulse was applied, calcein intensity decreased, indicating the efflux of calcein due to diffusion through transient holes on the cell membrane. Images were taken every 0.5 seconds. Playback is at 3.5 frames per second. No intensity modification was applied.

Reference

1 Medeiros, A. F. & Stepanishen, P. R. The forward and backward projection of acoustic fields from axisymmetric ultrasonic radiators using impulse response and Hankel transform techniques. *The Journal of the Acoustical Society of America* **75**, 1732-1740 (1984).
